# Supplementary material for: Multiplatform analyses reveal distinct drivers of systemic pathogenesis in adult versus pediatric severe acute COVID-19
Source: Nat Commun. 2023 Apr 4;14:1638. doi: 10.1038/s41467-023-37269-3 (PMC10073144; doi:10.1038/s41467-023-37269-3)
Supplement: Supplementary file 1 — Supplementary Information [file 41467_2023_37269_MOESM1_ESM.pdf]

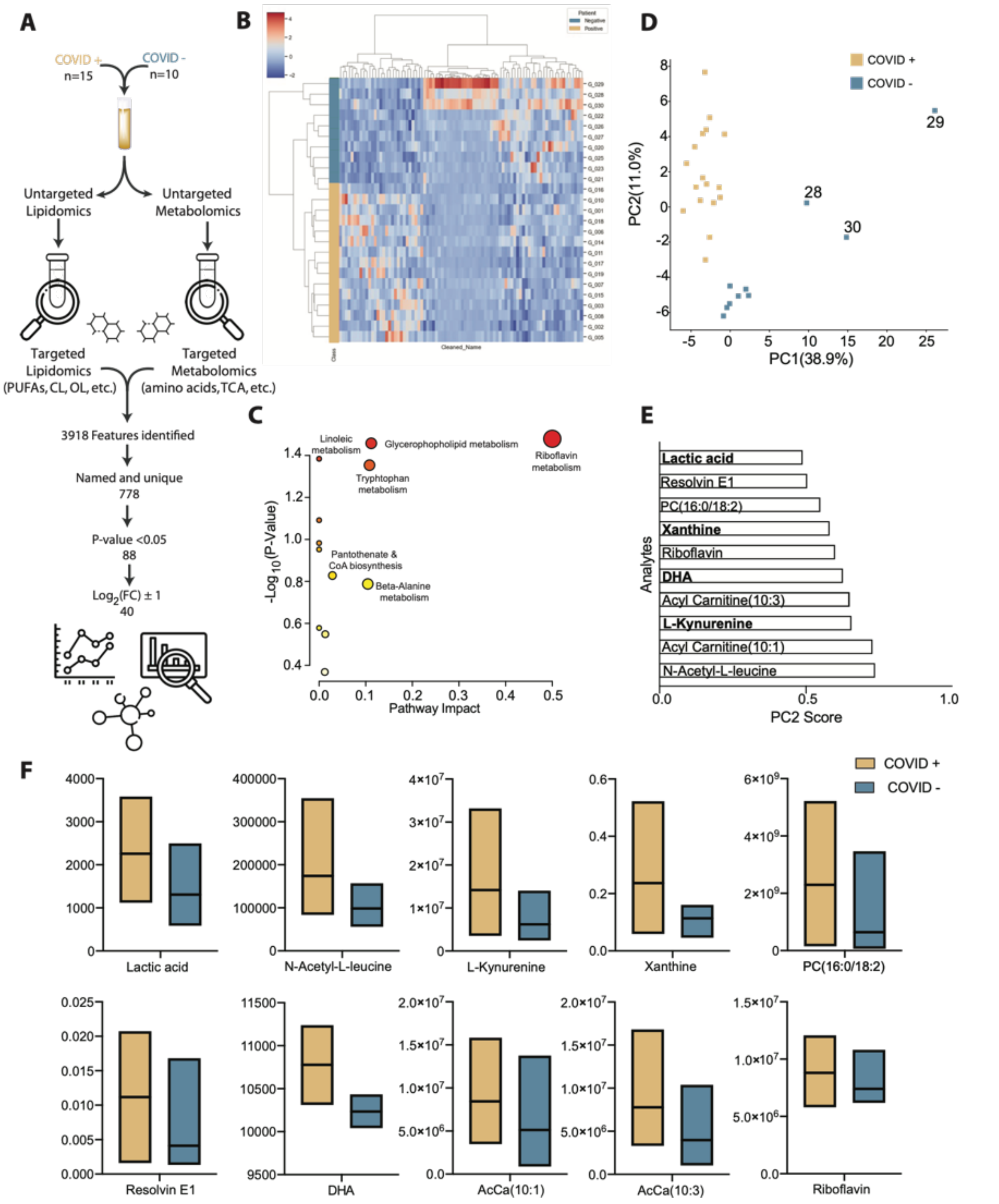

Supplementary Figure 1. Lipidomics and metabolomics analyses of adult cohort reveal alterations in Lactic acid, Xanthine, DHA and L-Kynurenine in COVID-19, consistent with findings from previous reports. A.) Workflow showing the targeted and untargeted assays used to interrogate lipid and metabolite changes occurring in COVID-19 patients (COVID+) compared to non-COVID patients (COVID-). B.) Heatmap with unbiased hierarchal clustering using significant analytes from COVID+ versus COVID- adults. C.) Bubble plot of analytes mapped to KEGG pathways using metaboanalyst (Fisher exact test). D.) Principal component analysis (PCA) using statistically significant metabolites. E.) and F.) Top 10 analytes that define separation of principal component two with abundances as determined by mass spectrometry analysis. Shown in box blots are the minima and maxima values as well as the mean of all measurements (COVID+ n=15; COVID- n=10). Source data are provided as a Source Data file.

Supplementary Table 1. Patient Demographics for Adult Multiomics Studies, n=25.

	COVID Cohort, n=15	Non-COVID Cohort, n=10
Age, median years (range)	70 (49 – 92)	65 (31 – 84)
Women, number (%)	7 (47%)	1 (10%)
Race, number (%)		
African American	12 (80%)	5 (50%)
White	3 (20%)	4 (40%)
Asian/Pacific Islander	0	1 (10%)
Body Mass Index, mean (SD)	33 (11)	29 (14)
Sample Time, mean number days post diagnosis (SD)	15 (12)	14 (13)
Illness Severity, median WHO COVID ordinal scale (range)	7 (5 – 8)	N/A
Non-COVID Underlying Diagnosis, number	N/A	
(%)		4 (40%)
Sepsis		5 (50%)
ARDS		1 (10%)
Cerebrovascular Accident		1 (10%)
Acute Leukemia		1 (10%)
Cardiogenic Shock		
Interventions, number (%)		
Renal-replacement therapy	7 (47%)	2 (20%)
Mechanical Ventilation	13 (87%)	5 (50%)
Vasopressor Support	9 (60%)	4 (40%)
Medications, number (%)		
Therapeutic Anticoagulation	11 (73%)	4 (40%)
Dexamethasone	1 (7%)	3 (30%)
Tocilizumab/Baricitinib/Remdesivir	0	0
Hydroxychloroquine	1 (7%)	0
Co-infections, number (%)		
Bacterial Pneumonia	1 (7%)	1 (10%)
Fungal Pneumonia	3 (20%)	2 (20%)
Urinary Tract Infection	1 (7%)	0
Clostridium difficile Colitis	1 (7%)	0
Co-morbidities		
Hypertension	11 (73%)	5 (50%)
Diabetes mellitus	9 (60%)	2 (20%)
Renal Disease	4 (27%)	3 (30%)
Obesity	6 (40%)	2 (20%)
Asthma or COPD	2 (13%)	4 (40%)
30 Day Disposition (%)		
Death/Hospice	5 (33%)	2 (20%)
Hospitalized/Transfer (inpatient rehab, long term acute care or other facility)	7 (47%)	1 (10%)
Home		
	3 (20%)	7 (70%)

Supplementary Dataset 1. Raw values for all unique features detected in lipidomic and metabolomic analyses for the adult cohort, available in the accompanying Source Data file.

Supplementary Dataset 2. Z normalized, knn imputed values for all unique features detected in lipidomic and metabolomic analyses for the adult cohort, available in the accompanying Source Data file.

Supplementary Dataset 3. Proteome discoverer table for adult proteomics experiments, available in the accompanying Source Data file.

Supplementary Table 2. Adult Proteomics Analysis Parameters.

Number of protein groups	625
Number of peptides	8,164
Numbers od PSM	127,056
Numbers of ms/ms spectra	1,471,228
Percentage miss cleavage=0	62.46%
Percentage miss cleavage=1	30.54%
Percentage miss cleavage=2	7%
PSM ΔM (PPM)	0.02 +/- 0.08

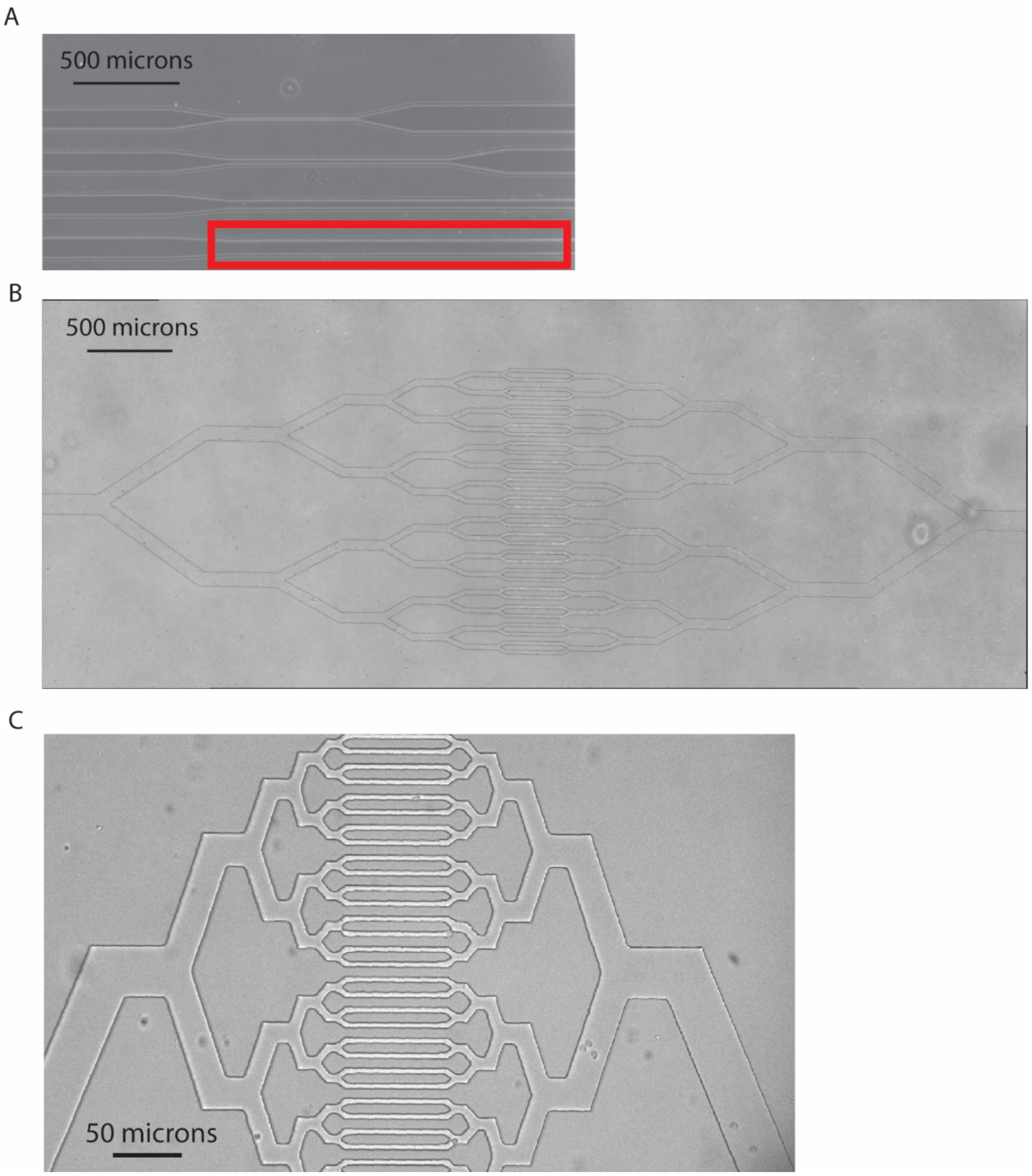

Supplementary Figure 2. Overview of Microfluidics Devices Used in Experiments. A.) Device used in aggregation assays consisting of parallel channels of varying sizes and uniform height of 10μm; the highlighted channel measures 70μm in width and was used in the described assays. B) Device used for endothelialized experiments with serial branches to a central channel width of 30μm and uniform 30μm height. C) Device used for single cell deformability assays with serial branches to a central channel width of 5μm and a uniform height of 6μm.

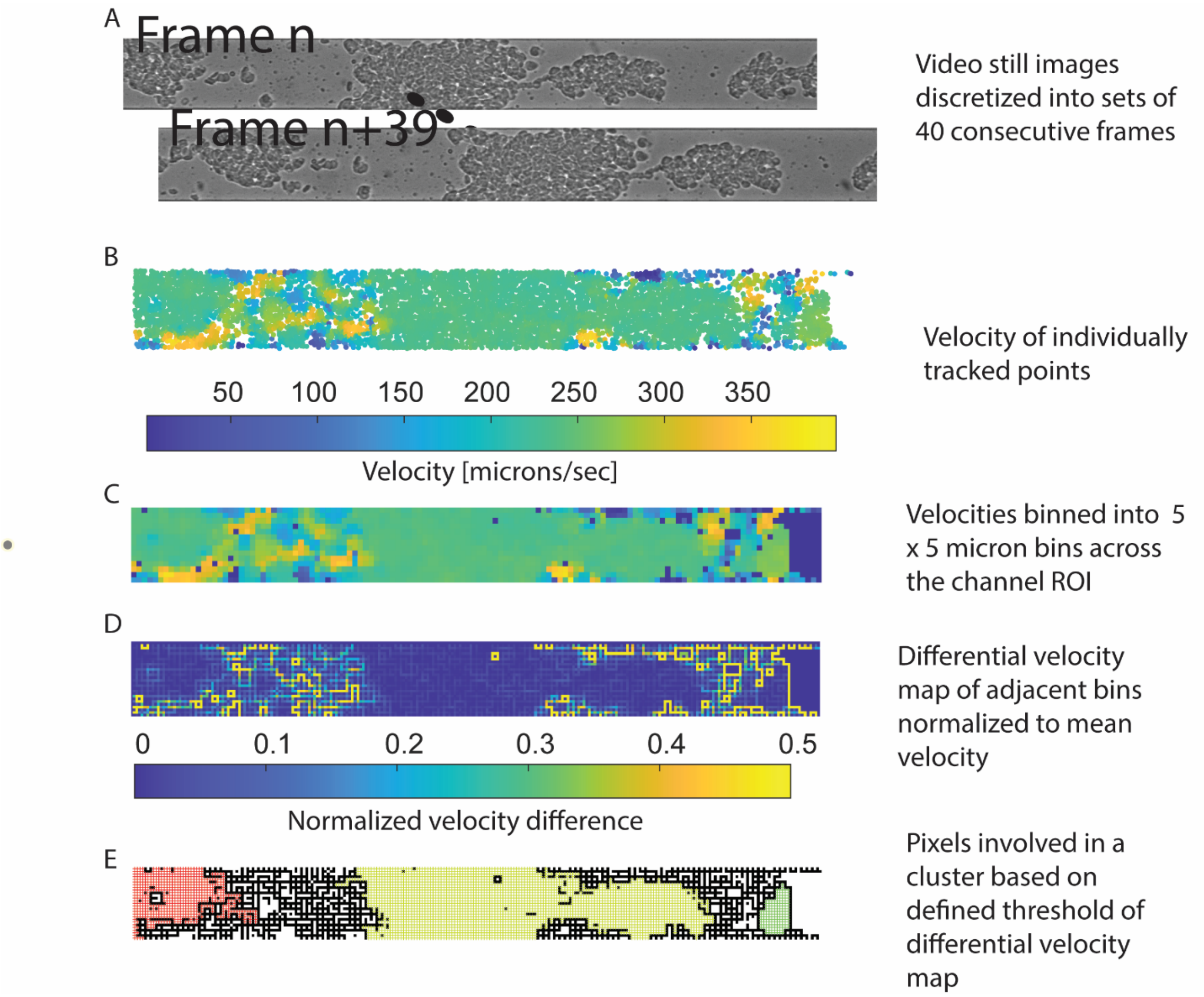

Supplementary Figure 3. Detailed Explanation of DCVC Quantification Workflow.
A.) Ten seconds of video microscopy was captured at a frame rate of 160 frames per second yielding 1600 frames per sample. This time resolved frame sample was discretized into 40 sets of 40 frames, corresponding to 0.25 seconds per set for cell feature tracking. B.) Individual cell features were detected in the first frame of each set and tracked over the subsequent 39 frames using a built-in Matlab (Mathworks) object which employs the Kanade-Lucas-Tomasi tracking algorithm. A cell feature was only included in the analysis if it was detected in all 39 frames. Instantaneous velocity was calculated from each frame step and then averaged over all frames within a set. C.) To account for the difference in the number of cell features tracked per unit area the velocity field was binned into a matrix with bin size 5 μm x 5μm, slightly smaller than the larger diameter of a single RBC. D.) The absolute difference in velocity between neighboring points in the binned velocity map were calculated and normalized to the average velocity for the set of images. E.) A black white image is created in which a threshold is applied to differential velocity map for a Boolean inclusion process of neighboring bins with <5% difference in velocity. Groups of connected points are then identified using a connectivity map and included as a DCVC (red, yellow, green) if they were at least the area of 15 RBCs which corresponded to 115 pixels in the image set. The size of each DCVC is calculated and mapped to the binned velocity matrix in (C) to calculate the aggregate velocity.

Supplementary Table 3. Patient Demographics for Adult Microfluidics Studies, n=29.

	COVID Cohort, n=13	Sepsis Cohort, n=16
Age, median years (range)	57 (24 – 84)	58 (19 – 81)
Women, number (%)	7 (54%)	4 (25%)
Race, number (%)		
African American	12 (92%)	12 (75%)
White	1 (8%)	4 (25%)
Asian/Pacific Islander	0 (0%)	0 (0%)
Body Mass Index, mean (SD)	33 (12)	28 (9)
Sample Time, mean number days post diagnosis (SD)	1.7 (0.7)	2.1 (0.7)
Illness Severity, median SOFA ordinal scale (range)	10 (3 – 19)	8.5 (5 – 15)
Interventions, number (%)		
Renal-replacement therapy	5 (38%)	3 (19%)
Mechanical Ventilation	10 (77%)	15 (94%)
Vasopressor Support	9 (69%)	14 (87%)
Medications, number (%)		
Therapeutic Anticoagulation	9 (69%)	3 (19%)
Dexamethasone	13 (100%)	0 (0%)
Tocilizumab/Baricitinib/Remdesivir	8 (62%)	0 (0%)
Infectious Source, number (%)		
Pneumonia	13 (100%)	8 (50%)
Bacteremia	0	5 (31%)
Urinary Tract Infection	0	2 (13%)
Skin and Soft Tissue Infection	0	1 (6%)
Co-morbidities, number (%)		
Hypertension	6 (46%)	8 (50%)
Diabetes mellitus	3 (23%)	5 (31%)
Renal Disease	2 (15%)	3 (19%)
Obesity	3 (23%)	5 (31%)
Asthma or COPD	3 (23%)	7 (43%)
30 Day Disposition, number (%)		
Death/Hospice	4 (31%)	8 (50%)
Hospitalized/Transfer (inpatient rehab, long term acute care or other facility)	5 (38%)	2 (13%)
Home	4 (31%)	6 (37%)

Supplementary Table 4. Patient Demographics for Pediatric Rheological Studies, n=25.

	COVID, n=11	MIS-C, n=14
Age, median years (range)	15 (6-20)	9.5 (5-19)
Girls, number (%)	7 (64%)	6 (43%)
Race/Ethnicity, number (%)		
African American/Non-Hispanic	4 (36%)	10 (71%)
African American/Hispanic	1 (9%)	0
White/Non-Hispanic	3 (27%)	0
White/Hispanic	1 (9%)	1 (7%)
Asian	2 (18%)	0
Declined/Non-Hispanic	0	1 (7%)
Declined/Hispanic	0	2 (14%)
Body Mass Index, mean (SD)	30 (14)	22 (8)
COVID or MIS-C Symptom Severity		
Severe, number (%)	11 (100%)	14 (100%)
Immunocompromised State, number (%)		
None	9 (82%)	13 (93%)
Malignancy	2 (18%)	1 (7%)
Medications		
Anticoagulation	8 (73%)	11 (79%)
Dexamethasone or other steroid	8 (73%)	12 (86%)
Tocilizumab/Baricitinib/Remdesivir	9 (82%)	2 (14%)
IVIG	2 (18%)	13 (93%)
Interventions		
Mechanical Ventilation	1 (9%)	3 (21%)
Vasopressor Support	3 (27%)	12 (86%)
ECMO	0	1 (7%)
Co-morbidities		
Obesity	5 (45%)	1 (7%)
Asthma	4 (36%)	2 (14%)
Peak Fibrinogen, normal 200-393 mg/dL		
Mean (SD)	512 mg/dL (161)	662 mg/dL (189)
Median (interquartile range)	485 mg/dL (422-630)	640 mg/dL (524-736)
Discharge Disposition (%)		
Death/Hospice	2 (18%)	1 (7%)
Home	9 (82%)	13 (93%)

Supplementary Table 5. Patient Demographics for Pediatric Multiomics Studies, n=19.

	COVID, n=7	MIS-C, n=5	Healthy, n=7
Age, median years (range)	15 (3-16)	8 (5-19)	9 (5-12)
Girls, number (%)	3 (43)	1 (20)	5 (71)
Race/Ethnicity, number (%)			
African American/Non-Hispanic	3 (43%)	4 (80%)	5 (71%)
White/Non-Hispanic	1 (14%)	0	2 (29%)
Declined/Hispanic	3 (43%)	1 (20%)	0
Body Mass Index, mean (SD)	26 (12)	24 (7)	Unknown
Sample Time, mean number days post diagnosis (SD)	4 (3)	6 (4)	N/A
COVID or MIS-C Severity, median WHO ordinal scale (range)	3 (2-5)	5 (4-7)	N/A
Immunocompromised State, number (%)			N/A
None	4 (57%)	4 (80%)	
Malignancy	3 (43%)	1 (20%)	
Medications			N/A
Anticoagulation	2 (29%)	3 (60%)	
Dexamethasone or other steroid	1 (14%)	3 (60%)	
Tocilizumab/Baricitinib/Remdesivir	3 (43%)	2 (40%)	
IVIG	2 (29%)	1 (20%)	
Interventions			N/A
Mechanical Ventilation	0	2 40%)	
Vasopressor Support	1 (14%)	4 (80%)	
ECMO	0	0	
Co-morbidities			N/A
Obesity	3 (43%)	0	
Asthma	2 (29%)	1 (20%)	
Immunosuppressive therapy	3 (43%)	1 (20%)	
Co-infections			N/A
Acute appendicitis	1 (14%)	0	
Discharge Disposition (%)			N/A
Death/Hospice	1 (14%)	1 (20%)	
Home	6 (86%)	4 (80%)	

A

Number of protein groups	925
Number of peptides	8,075
Number of PSM	104,789
Number of ms/ms spectra	916,220
Percentage miss cleavage=0	68.52%
Percentage miss cleavage=1	25.73%
Percentage miss cleavage=2	4.9%
PSM ΔM (PPM)	0.04 +/- 1.0

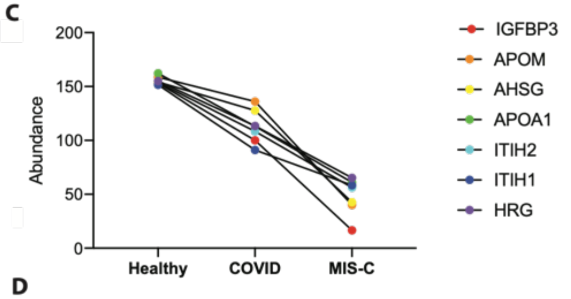

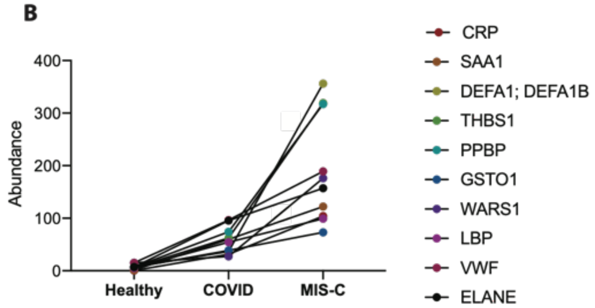

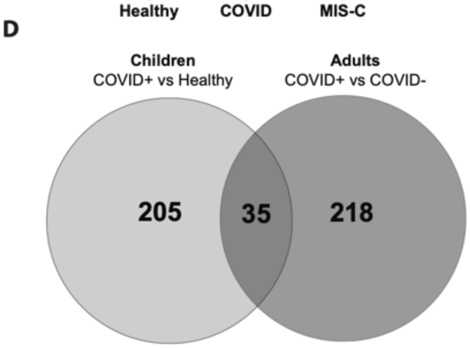

Supplementary Figure 4. Pediatric analyte analysis. A.) Table depicting the parameters of our pediatric cohort mass spectrometry run from number of proteins to the percentage of cleavages and average mass error (ΔM) (parts per million (PPM)). B.) & C.) Proteins identified that trend with disease and could be diagnostic for alterations in MIS-C and COVID-19 in children. D.) Venn diagram of the significantly altered proteins (FDR <0.01), lipids and metabolites (p-value <0.05) when comparing the adult cohort (COVID+ versus (vs) COVID-) with the pediatric cohort (COVID+ vs Healthy). Analytes from (D) are listed individually in Supplementary Dataset 11. Source data are provided as a Source Data file.

Supplementary Dataset 4. Proteome discoverer table for pediatric proteomics experiments, available in the accompanying Source Data file.

Supplementary Dataset 5. Raw values for all unique features detected in lipidomic and metabolomic analyses for the pediatric cohort, available in the accompanying Source Data file.

Supplementary Dataset 6. Z normalized, knn imputed values for all unique features detected lipidomic and metabolomic analyses for the pediatric cohort, available in the accompanying Source Data file.

Supplementary Dataset 7. Edged analysis for heat map of adult cohort, available in the accompanying Source Data file.

Supplementary Dataset 8. Edged analysis for heat map of pediatric cohort, available in the accompanying Source Data file.

Supplementary Dataset 9. Centrality analysis used to generate network plots of the adult cohort, available in the accompanying Source Data file.

Supplementary Dataset 10. Centrality analysis used to generate network plots of the pediatric cohort, available in the accompanying Source Data file.

Supplementary Table 6. Chromatography parameters for untargeted lipidomics.

Time (min)	A: 60:40 ACN:H2O	B: 90:10 IPA:ACN
0	60	40
0.2	60	40
1.5	40	60
6	30	70
9	15	85
11	0	100
12.5	0	100
13	60	40
15	60	40

Supplementary Table 7. Mass spectrometry parameters for untargeted lipidomics.

Parameter	Setting
Ion Source Type	H-ESI
Spray Voltage	Static
Positive Ion (V)	3500 V
Negative Ion (V)	3500 V
Sheath Gas (arb. units)	50
Aux Gas (arb. units)	10
Sweep Gas (arb. units)	1
Ion Transfer Tube Temp (°C)	300
Capillary Temp (°C)	275
S-Lens RF Level	40V
Data Acquisition Strategy	Full scan and data dependent acquisition
Full scan resolution (FWHM)	120,000
MS/MS resolution (FWHM)	30,000
HCD Fragmentation:	Normalized collision energy (NCE) of 25, 35, 45, 55 eV
	Stepped NCE of 10, 20, 50 eV

Supplementary Table 8. Lipid view parameters.

**Identification**
Database
• Target database: General, HCD, CID, Labeled GPL, GL, SP, ChE
Peak Detection
• Recalc Isotope: On
• R.T. Interval (min): 0.5
• R.T. Range (min): NA
Search Options
• ProductSearch_QEX
• SearchType: Product
• ExpType: LC
• Precursor Tol: 5.0 ppm
• Product Tol: 5.0 ppm
• Intensity Threshold: Relative; Product Ion 1.0 %
• m-Score Threshold: 2.0
**Quantitation**
Execute Quantitation: On
Mz Tol: -0.5, +0.5
Tol Type: Da
RT range (min): -0.1, +0.1
**Filters**
Top Rank Filter: On

Main node filter: All isomer peaks
m-Score Threshold (Display): 5.0
FA Priority: On
ID Quality Filter: A,B,C,D
**Class**
Phospholipids:
• CL, Cardiolipin
• LPA, Lysophosphatidic Acid
• PA, Phosphatidic Acid
• LPC, Lysophosphatidylcholine
• PC, phosphatidylcholine
• LPE, Lysophosphatidylethanolamine
• PE, Phosphatidylethanolamine
• LPG, Lysophosphatidylglyercol
• PG, Phosphatidylglycerol
• LPI, Lysophosphatidylinositol
• PI, Phosphatidylinositol
• LPS, Lysophosphatidylserine
• PS, Phosphatidylserine
• PIP, Phosphatidylinositol
• PIP2, Phosphatidylinositol
• PIP3, Phosphatidylinositol
Sphingolipids:
• Cer, Ceramides
• CerP, Ceramides Phosphate
• CerPE, Ceramide Phosphoethanolamines
• Hex1Cer, Simple Glc Series
• Hex2Cer, Simple Glc Series
• Hex3Cer, Simple Glc Series
• LSM, Lysosphingomyelin
• SM, Sphingomyelin
• SPH, Sphingosine
• SPHP, Sphingosine phosphate
Neutral Lipids:
• ChE, Cholesterol Ester
• DG, Diglyceride
• MG, Monoglyceride
• TG, Triglyceride
Fatty Acyl and Other Lipids:
• AcCa, Acyl Carnitine
• Co, Coenzyme

• FA, Fatty Acid
• PAF, Platelet Activating Factor
• AcylCoA, Acyl-coenzyme A
Fatty Acyl Carnitines:
• CarE, Carnitine Esters
**Ion**
Adducts:
Negative:
• -H
• +HCOO
• +CH3COO
• -2H
• -CH3
Positive:
• +H
• +NH4
• +Na
• +H-H2O
• +H-2H2O
• +2H

**Alignment**
Search Type: Product
Exp Type: LC-MS
Alignment Method: Mean
RT Tolerance: 0.1
Rate unassigned peak area: On
Filter Type: New Filter
Top rank Filter: On
Main Node Filter: All isomer peaks
m-Score Threshold: 5.0
ID Quality Filter: A,B,C,D

Supplementary Table 9. Lipid search parameters.

**LipidView Analysis Method Details**
Method Name = PrecQTrap
APPLIES TO: QTRAP
DATA:
Data Processing: NL,PS
Polarity = Negative
Combine Multiple Periods & Exp's: USE
MS/MS: Extract MS/MS Data
SPECTRUM:
NL,PS: Mass Tolerance = 0.5, Min % Intensity = 0.1, Minimum S/N = 3
Mass Tolerance = 0.5, Min % Intensity = 0.1, Minimum S/N = 3
MS/MS: Fragments File = Fragments_neg, Mass Tolerance (Da) = 0.5, Spectrum Peaks > Noise* 3, Avg. Repl. Data: USE
Search m/z Range from 400 to 1200
Deisotope: USE
Liquid Chromatography: Average Spectrum from 1 to 20 minutes
LIPID DETAILS:
Processing Method = Identify Species, Report Unidentified Peaks: USE
Analysis Types:
Glycerophospholipids, Sphingolipids
Selected classes for each used analysis type:
Glycerophospholipids:
PA, PC, PE, PG, PI, PS, CL, MMPE, DMPE
Total Double Bonds <= 12
Lysospecies: USE
Sphingolipids:
SM, Cer
Total Double Bonds <= 12
Nbr of OH groups: From 3 To 4

Supplementary Table 10. Chromatography parameters for polyunsaturated fatty acids.

Time (min)	60:40 ACN:Water	90:10 IPA: ACN
0	80	20
2.0	80	20
2.1	60	40
10.0	30	70
10.1	0	100
15.0	0	100
15.1	80	20
18.0	80	20

Supplementary Table 11. Mass spectrometry parameters for polyunsaturated fatty acids.

	Negative polarity
Curtain gas	20 arb. units
Collision Gas	Low
IonSpray Voltage	-4500 V
Temperature	650C
Ion Source Gas 1	55 arb. units
Ion Source Gas 2	60 arb. units
Declustering Potential	-90 arb. units
Entrance Potential	-10eV
Collision Energy	-30eV
Collision Cell Exit Potential	-11eV

Supplementary Table 12. Gradient parameters for chromatography analysis of oxylipins.

Time (min)	Percent A: 0.1% FA in water	Percent B: 0.1% FA in ACN
0	90	10
0.5	90	10
1	50	50
2	50	50
2.1	25	75
5	25	75
7	15	85
12	15	85
12.1	0	100
14	0	100
15	90	10
16	90	10

Supplementary Table 13. Gradient parameters for chromatography analysis of endocannabinoids.

Time (min)	Percent A: 0.1% FA in water	Percent B: 0.1% FA in ACN
0	90	10
0.5	90	10
1	50	50
2	50	50
2.1	15	85
5	15	85
7	15	85
12	15	85

12.1	90	10
13.0	90	10

Supplementary Table 14. Mass spectrometry parameters for acquisition of oxylipins and endocannabinoids.

	Negative polarity	Positive polarity
Curtain gas	20	20
Collision Gas	Low	Low
IonSpray Voltage	-4500	5000
Temperature	650	650
Ion Source Gas 1	60	55
Ion Source Gas 2	50	60
Declustering Potential	-200	90
Entrance Potential	-10	10
Collision Energy	-40	47
Collision Cell Exit Potential	-11	18

Supplementary Table 15. Mass spectrometry analysis of oxylipins and endocannabinoids.

LIPID	Q1 MASS	Q3 MASS
9,10 DIHOME	313.2	201.0
PGE E2	351.2	315.1
20- HETE	319.2	289.2
9-HETE	319.2	167.2
14,15 DHET	337.2	207.0
5 HETE	319.2	115.1
12 R-HETE	319.2	179.1
11,12-DHET	337.2	167.1
8,9-DHET	337.2	127.2
5,6 EET	319.2	191.1
5,6-DHET	337.2	71.0
TXB2	369.2	169.1
12(13)-EPOME	295.2	195.1
13 HODE	295.2	195.1
PGF2A	353.2	193.3
14(15)-EET / 15-HETE	319.2	219.0
LTB4	335.2	195.1
8(9)-EET	319.2	69.2
11(12)-EET	319.2	167.1
PGE2 Ethanolamide (PGE2-EA)_271.3	396.5	271.3
Oleoyl Ethanolamide (OEA)	326.4	62.1
Palmitoyl Ethanolamide	300.4	62.1
ARA-Ethanolamide (AEA)	348.4	62.1
Docosahexaenoyl Ethanolamide (DHEA)	372.4	62.1

Linoleoyl Ethanolamide (LEA)	324.4	62.1
Stearoyl Ethanolamide (ceramid)	328.4	62.1
oxy-Arachidonoyl Ethanolamide (oxy-AEA)	364	76
2-Arachidonoyl Glycerol (2AG) trans1	379.4	135
Docosatetraenoyl Ethanolamide (DEA)	376.59	62.1
alpha-linolenoyl ethanolamide(ALEA)	322.4	62.1
oleamide	282.5	247.4
dihomo-gamma-linolenoyl ethanolamide	350.4	62.1
docosanoyl ethanolamide	384.5	62.1

Supplementary Table 16. Chromatography parameters for untargeted metabolomics.

Time (min)	Solvent A: 0.1% FA in Water	Solvent B: 0.1% FA in ACN	Flow Rate (ml/min)
-4.0	10	90	0.35
0	10	90	0.25
17.5	80	20	0.25
23.0	80	20	0.35
24.0	10	90	0.35

Supplementary Table 17. Parameters used for data acquisition analysis in untargeted metabolomics.

Sheath Gas	40 arb. units	Electrospray Voltage (Positive)	3000 V
Aux Gas	8 arb. units	Electrospray Voltage (Negative)	2750 V
Sweep Gas	1 arb. units	Capillary Temp.	275 ˚C
RF Level	50	Probe Temp.	320 ˚C

Supplementary Table 18. Compound discoverer parameters.

		ADULT POS	ADULT NEG	PEDS POS	PEDS NEG
VERSION				3.2.0.421	3.2.0.421
GENERAL	SN Thresh	3	3	3	3
	Min Peak Int	200k	200k	200k	200k
	Max Peak Wid	0.5 min	0.5 min	0.5 min	0.5 min
	Min # Scans/Peak	5	5	5	5
GROUP CPDS	Mass Tol	5 ppm	5 ppm	5 ppm	5 ppm
	RT Tol	0.2 min	0.2 min	0.2 min	0.2 min
FILL GAPS	SNThresh	1.5	1.5	1.5	1.5
	Use Real Peak Detection	TRUE	TRUE	TRUE	TRUE
QC CORRE	Reg	Linear	Linear	Linear	Linear
	Min QC Cov	75	75	75	75
	Max QC RSD	50	50	50	50

	Max Corr QC RSD	30	30	30	30
	Files Between	20	20	20	20
**MARK BGD**	Max Sample/Blank	5	5	5	5
**MASS LISTS**	Mass Tol	7 ppm	7 ppm	5 ppm	5 ppm
	Use RT	TRUE	TRUE	TRUE	TRUE
	RT Tol	1 min	1 min	1 min	1 min
**SEARCH MZCLOUD**	Match Factor Thresh	60	60	60	60

Supplementary Dataset 11. Overlap between differentially abundant species identified in Adult and Pediatric cohorts, available in the accompanying Source Data file.

Additional Supplementary Data is provided in the accompanying Source Data file.
