# Supplementary material for: Multiplatform analyses reveal distinct drivers of systemic pathogenesis in adult versus pediatric severe acute COVID-19
Source: Nat Commun. 2023 Apr 4;14:1638. doi: 10.1038/s41467-023-37269-3 (PMC10073144; doi:10.1038/s41467-023-37269-3)
Supplement: Supplementary file 3 — Reporting Summary [file 41467_2023_37269_MOESM3_ESM.docx]

1

nature portfolio | reporting summary

*March 2021*


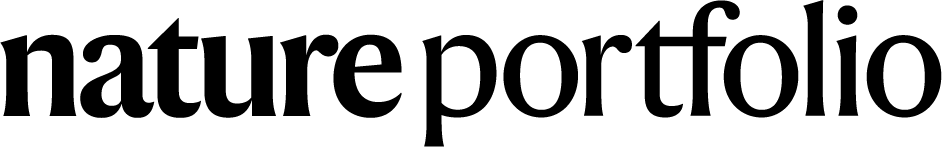
Corresponding author(s): Maier, Ortlund and Lam

Last updated by author(s): Mar 3, 2023

Reporting Summary

Nature Portfolio wishes to improve the reproducibility of the work that we publish. This form provides structure for consistency and transparency in reporting. For further information on Nature Portfolio policies, see our Editorial Policies and the Editorial Policy Checklist.

Please do not complete any field with "not applicable" or n/a. Refer to the help text for what text to use if an item is not relevant to your study. For final submission: please carefully check your responses for accuracy; you will not be able to make changes later.

## Statistics

For all statistical analyses, confirm that the following items are present in the figure legend, table legend, main text, or Methods section. n/a Confirmed


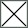


The exact sample size (*n*) for each experimental group/condition, given as a discrete number and unit of measurement

A statement on whether measurements were taken from distinct samples or whether the same sample was measured repeatedly The statistical test(s) used AND whether they are one- or two-sided

*Only common tests should be described solely by name; describe more complex techniques in the Methods section.*

A description of all covariates tested

A description of any assumptions or corrections, such as tests of normality and adjustment for multiple comparisons

A full description of the statistical parameters including central tendency (e.g. means) or other basic estimates (e.g. regression coefficient) AND variation (e.g. standard deviation) or associated estimates of uncertainty (e.g. confidence intervals)

For null hypothesis testing, the test statistic (e.g. *F*, *t*, *r*) with confidence intervals, effect sizes, degrees of freedom and *P* value noted

*Give P values as exact values whenever suitable.*

For Bayesian analysis, information on the choice of priors and Markov chain Monte Carlo settings

For hierarchical and complex designs, identification of the appropriate level for tests and full reporting of outcomes Estimates of effect sizes (e.g. Cohen's *d*, Pearson's *r*), indicating how they were calculated

*Our web collection on statistics for biologists contains articles on many of the points above.*

## Software and code

Policy information about availability of computer code Data collection

RBC aggregation was quantified using a commercially available video tracking algorithm and additional custom software. Specifically, individual cell features were detected using a built-in Matlab (Mathworks) object which employs the Kanade-Lucas-Tomasi tracking algorithm. A cell feature was only included in the analysis if it was detected in all 39 frames. Instantaneous velocity was calculated from each frame step and then averaged over all frames within a set. To account for the difference in the number of cell features tracked per unit area the velocity field was binned into a matrix with bin size 5 µm x 5µm, slightly smaller than the larger diameter of a single RBC. The absolute difference in velocity between neighboring points in the binned velocity map were calculated and normalized to the average velocity for the set of images. A black white image is created in which a threshold is applied to differential velocity map for a Boolean inclusion process of neighboring bins with <5% difference in velocity. Groups of connected points are then identified using a connectivity map and included as a RBC cluster (red, yellow, green) if they were at least the area of 15 RBCs which corresponded to 115 pixels in the image set. The size of each RBC cluster is calculated and mapped to the binned velocity matrix in to calculate the aggregate velocity.

Data analysis

Proteomics data was analyzed using the commercially available software Proteome Discoverer version 2.4. Lipid identification was performed by uploading raw mass spectral data into LipidSearch software version 4.2. Pathway analysis was performed using the Kyoto Encyclopedia of Genes and Genomes [(https://www.genome.jp/kegg/pathway.html).](http://www.genome.jp/kegg/pathway.html)) For metabolite and lipidomics analyses, data from each assay were independently analyzed using the R package, xmsPANDA (https://github.com/kuppal2/xmsPANDA). The R package xMWAS (https:// github.com/kuppal2/xMWAS) was used to integrate cytokines and metabolomics data. Pathway analysis was performed using MetaboAnalyst version 5.0. All code related to microfluidics raw data post-processing workflow can be found at https://github.com/lmiffrig/NatCommCOVID.

For manuscripts utilizing custom algorithms or software that are central to the research but not yet described in published literature, software must be made available to editors and reviewers. We strongly encourage code deposition in a community repository (e.g. GitHub). See the Nature Portfolio guidelines for submitting code & software for further information.

## Data

Policy information about availability of data

All manuscripts must include a data availability statement. This statement should provide the following information, where applicable:

- Accession codes, unique identifiers, or web links for publicly available datasets
- A description of any restrictions on data availability
- For clinical datasets or third party data, please ensure that the statement adheres to our policy

Data reported in this study are publicly available. The mass spectrometry proteomics data have been deposited to the ProteomeXchange Consortium via the PRIDE partner repository with project accession PXD040437 and project DOI 10.6019/PXD040437, and project accession PXD040438 and project DOI 10.6019/PXD040438. MS/MS spectra in the study were searched using uniport taxon ID9606 [(https://www.uniprot.org/taxonomy/9606).](http://www.uniprot.org/taxonomy/9606)) The lipidomics, metabolomics, and microfluidics data are provided in the Supplementary Information/Source Data files. All other source data are provided with this paper in the accompanying Source Data file.

## Human research participants

Policy information about studies involving human research participants and Sex and Gender in Research.

Reporting on sex and gender

Gender was determined based on clinical chart review and was not specifically considered in the study design. Source data disaggregated for gender is not provided as consent for individual-level reporting was not obtained.

Population characteristics

Demographic information for all patients was obtained by electronic chart review. For adult multiomics studies a convenience sample of 15 COVID-positive (COVID) and 10 COVID-negative (non-COVID) critically ill patients were included with the following characteristics: COVID group: median age 70 (range 49-92), 47% women, 80% african american and 20% white, mean body mass index 33 (11 std dev); non-COVID group: median age 65 (range 31-84), 10% women, 50% african american, 40% white and 10% asian/pacific islander, mean body mass index 29 (14 std dev). For adult microfluidics studies prospectively enrolled patients meeting sepsis criteria included 13 COVID-positive and 16 COVID-negative patients with the following characteristics: COVID group: median age 57 (range 24-84), 54% women, 92% african american and 8% white, mean body mass index 33 (12 std dev), median SOFA score 10 (range 3-19), 46% with hypertension, 23% with diabetes, 15% with renal disease, 23% with obesity, 23% with asthma or COPD; non-COVID group: median age 58 (range 19-81), 25% women, 75% african american and 25% white, mean body mass index 28 (9 std dev), median SOFA score 8.5 (range 5-15) 50% with hypertension, 31% with diabetes, 19% with renal disease, 31% with obesity, 43% with asthma or COPD. Pediatric multiomics studies included the following: acute COVID: n=7, median age 15 (range 3-16), 43% girls, 43% african american/ non-hispanic, 14% white/non-hispanic, 43% declined/hispanic, mean body mass index 26 (st dev 12), 43% with obesity, 29% with asthma, 43% with immunosuppressive therapy; MIS-C: n=5, median age 8 (range 5-9), 20% girls, 80% african american/ non-hispanic, 0% white/non-hispanic, 20% declined/hispanic, mean body mass index 24 (st dev 7), 0% with obesity, 20% with asthma, 20% with immunosuppressive therapy.

Recruitment

All human samples were obtained in compliance with Emory’s Institutional Review Board determination. Patients or their legal representatives were approached for study inclusion. Patients consenting to participation in this study were not compensated.

Ethics oversight

All studies were conducted according to the guidelines of the Declaration of Helsinki and approved by the Institutional Review Board (IRB, or Ethics Committee) of Emory University (IRB00000723, IRB00087446, STUDY00000401, and STUDY00000510)

Note that full information on the approval of the study protocol must also be provided in the manuscript.

# Field-specific reporting

Please select the one below that is the best fit for your research. If you are not sure, read the appropriate sections before making your selection.

Life sciences

Behavioural & social sciences

Ecological, evolutionary & environmental sciences

For a reference copy of the document with all sections, see nature.com/documents/nr-reporting-summary-flat.pdf

2

nature portfolio | reporting summary

*March 2021*

# Life sciences study design

No statistical method was used to predetermine sample size. Multiomics analyses on a limited initial cohort were meant to be hypothesis- generating and allowed for identification of specific changes and differential regulation supported by previous studies, suggesting that, despite a limited initial cohort, our methodology and results are robust in terms of biological and statistical significance. Our goal with this cohort was to identify pathways potentially involved in disease pathogenesis that could be further and more directly investigated through detailed mechanistic studies. To this end, we performed a robust mechanistic investigation of altered blood rheology and red blood cell aggregation using microfluidic assays in a well-balanced and well-powered clinical cohort that was matched for baseline characteristics between COVID-positive and COVID-negative patients.

All studies must disclose on these points even when the disclosure is negative. Sample size

3

nature portfolio | reporting summary

*March 2021*

Data exclusions

No data were excluded from analyses.

Replication

All studies were replicated for reproducibility to the extent possible and demonstrated consistent findings. Microfluidics studies were repeated at least 3 times with similar results. Biomarker analyses were performed in duplicate. Representative micrograph images are from one of 6 independent autopsies.

Randomization

Samples were allocated based on underlying diagnosis, thus randomization is not applicable.

Blinding

All samples were de-identified prior to receipt for proteomics, lipidomics and metabolomics experiments, and subsequent experimentation and analysis were blinded.

# Reporting for specific materials, systems and methods

We require information from authors about some types of materials, experimental systems and methods used in many studies. Here, indicate whether each material, system or method listed is relevant to your study. If you are not sure if a list item applies to your research, read the appropriate section before selecting a response.

Materials & experimental systems Methods


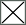

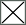

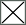

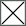


n/a Involved in the study Antibodies Eukaryotic cell lines

Palaeontology and archaeology Animals and other organisms Clinical data

Dual use research of concern


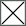

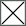

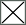


n/a Involved in the study

ChIP-seq

Flow cytometry

MRI-based neuroimaging

## Antibodies

Antibodies used

Enzyme-linked immunoassay kits were used to detect biomarkers syndecan-1 (Abcam, ab46506) and von Willebrand factor (Abcam, ab223864) in patient plasma following the manufacturer's instructions. There was no experimental use of antibodies in the study.

Validation

Non-COVID sepsis patients (positive control) and healthy volunteer (negative control) samples were run in parallel with experimental COVID patient samples, and demonstrated expected results. In addition, the procedure followed the manufacturer's (Abcam) instructions exactly.

## Eukaryotic cell lines

Policy information about cell lines and Sex and Gender in Research Cell line source(s)

Human umbilical vein endothelial cells (HUVECs, Lonza, CC-2519) were cultured using a well-established protocol.

Authentication

Primary cells were used between passages 4-7 and demonstrated typical morphological and phenotypic characteristics.

Mycoplasma contamination

Cells were not tested for mycoplasma contamination.

Commonly misidentified lines

Not applicable.

(See ICLAC register)


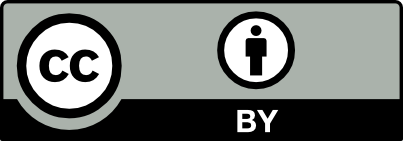
This checklist template is licensed under a Creative Commons Attribution 4.0 International License, which permits use, sharing, adaptation, distribution and reproduction in any medium or format, as long as you give appropriate credit to the original author(s) and the source, provide a link to the Creative Commons license, and indicate if changes were made. The images or other third party material in this article are included in the article's Creative Commons license, unless indicated otherwise in a credit line to the material. If material is not included in the article's Creative Commons license and your intended use is not permitted by statutory regulation or exceeds the permitted use, you will need to obtain permission directly from the copyright holder. To view a copy of this license, visit <http://creativecommons.org/licenses/by/4.0/>
